# Radiocesium Concentration in Commercially-Available Foods Produced in Japan: 2017-2019

**DOI:** 10.14252/foodsafetyfscj.D-21-00011

**Published:** 2022-03-08

**Authors:** Hiromi Nabeshi, Masataka Imamura, Tomoaki Tsutsumi, Tomomi Maeda, Akiko Hachisuka, Hiroshi Akiyama

**Affiliations:** 1 Division of Foods, National Institute of Health Sciences, 3-25-26 Tonomachi, Kawasaki-ku, Kawasaki, Kanagawa 210-9501, Japan; 2 Division of Biochemistry, National Institute of Health Sciences, 3-25-26 Tonomachi, Kawasaki-ku, Kawasaki, Kanagawa 210-9501, Japan; 3 Present address: Department of Analytical Chemistry, Faculty of Pharmaceutical Sciences, Hoshi University, 2-4-41 Ebara, Shinagawa-ku, Tokyo 142-8501, Japan

**Keywords:** commercially-available foods, cultivation/feeding control, natural food, radioactive cesium, screening inspection

## Abstract

We investigated the concentration of radioactive cesium (r-Cs: ^134^Cs and ^137^Cs) in commercially-available foods to confirm the effectiveness of pre-shipment radioactive material inspections mainly conducted by local governments. We focused on selected production areas and foods with high probability of r-Cs detection. To this end, we evaluated 715, 685, and 683 samples using scintillation spectrometer and high-purity germanium γ-spectrometer in fiscal years 2017, 2018, and 2019, respectively. The results accounted for 9 samples (1.3%), 10 samples (1.5%), and 5 samples (0.7%) for each fiscal year exceeded the standard limit of radioactive material (100 Bq/kg as r-Cs concentration for general foods). Although we selected and evaluated foods with high probability of r-Cs detection, percentage of samples exceeding the standard limit in each fiscal year was very low, less than 2% to be exact. This suggests that food management system, including pre-shipment inspections, were effectively functioning. In addition, samples exceeding the standard limit were bound to edible wild plants and wild mushrooms, and log-cultivated mushrooms. The former is consider to be difficult for cultivation/feeding control, and the latter was know to be parts of foods greatly affected by radioactive materials. This suggests that the concentration of r-Cs in these items remains at relatively high levels. In contrast, r-Cs was not detected in items with controalble cultivation/feeding. Based on these observations, it is better to be inspected on more difficult-to-cotrol cultivation/feeding items, in order to achieve further streamlining and improving of inspection efficiency. Our results indicate that r-Cs concentration in commercially-available foods of easy-to cultivation/feeding control, such as general vegetables, fruits, and meat, have been well-controlled in Japan, however, difficult-to-cultivation/feeding control items need to be more paid attention to r-Cs concentrations.

## Introduction

The Ministry of Health, Labour and Welfare (MHLW) has established provisional regulatory values for radioactive materials in foods. This is in response to the radioactive contamination of foods caused by the accident at Tokyo Electric Power Company’s Fukushima Daiichi Nuclear Power Plant (FDNPP) in March 2011. Local governments also formulated their own inspection plans, conducted pre-shipment inspections of radioactive materials, mainly radioactive cesium [r-Cs] and iodine, and imposed restrictions on shipments based on the inspection results, contributing to prevent distributions of foods with radioactive materials exceeding provisional regulatory values^1^^)^. In fiscal 2011, we purchased commercially-available foods and surveyed their concentration of r-Cs (the sum of ^134^Cs and ^137^Cs) to confirm the effectiveness of pre-shipment radioactive material inspections^2^^)^. The results have shown that 6 out of 1,427 samples (0.4%) exceeded the provisional regulatory value of 500 Bq/kg (the value for foods except drinking water, milk, and dairy products). This confirmed that the pre-shipment radioactive material inspections, conducted by the local governments, were functioning properly.

The current standard limits (100 Bq/kg as r-Cs concentration for general foods) were enforced in April 2012 to further ensure the food safety^3^^)^. This is a stricter standard than the provisional regulatory values. There have been some reports published by local governments on r-Cs concentrations of commercially-available foods after the enforcement of current standard limits^4^^,^^5^^,^^6^^)^. We have been also investigating commercially-available foods, taking account in the results from previous inspections. Our surveys were done in fiscal years of 2012 and 2013. The proportion of samples that exceeded the standard limit was 0.2% in both fiscal years among all the examined samples.their results were lower than results of fiscal 2011 survey even though that the standard limit had been lowered ever since^7^^)^. These results confirmed that both the effectiveness of pre-shipment inspections based on the lowered standard limit and of the shipping restrictions. Between fiscal 2014 and 2016, we surveyed the purchased products mainly consisting of known food items and areas with high probability of r-Cs detection^8^^)^. The percentage of samples that exceeded the standard limit was about 1% in that survey, although those food itemss were selectd from those with high r-Cs detection probability, such as wild mushrooms, log-cultivated mushrooms, and edible wild plants harvested in the Tohoku and Northern Kanto areas. This confirmed that the systems of pre-shipment inspections and shipment restrictions by local governments were established properly and functioning effectively. Nevertheless, there were some samples of wild mushrooms, edible wild plants, game meat, and log-cultivated mushrooms found to be with high r-Cs concentrations, ithus continuous monitoring is needed.

Accumulated inspection results and contamination reduction measures are subjected for continuous reviews in accordance with the “Concepts of Inspection Planning and the Establishment and Cancellation of Items and Areas to which Restriction on Distribution and/or Consumption of Foods concerned Applies (27 June, 2011)”^9^^)^. This provides guidelines stipulating basic matters such as inspection planning and shipment restrictions. The decline in number of items exceeding the standard limit in recent inspections lead to a review of the guidelines in March 2017 for items that were considered to be either possible or difficult to control cultivation/feeding control^10^^)^. It was assessed wheather improvement of inspection efficiency is possible mainly in items of possible cultivation/feeding control.

To verify the effectiveness of pre-shipment radioactive material inspection following the establishment of items, fregardless of easiness of cutivation/feeding control, we surveyed r-Cs concentrations in commercially-available foods between fiscal 2017 and 2019, included in this study. Based on the previous reports’ trend that r-Cs concentrations was detected in in edible wild plants, wild mushrooms, log-cultivated mushrooms, the items cultivation/feeding control is difficult with, were especially focused in this study. For items of easy-to-control cultivation/feeding. This study focused inspectuion on those based on the plans formulated by local governments, especially for items with decreased the number of inspections in comparison to fiscal 2016, a year before revision of the guidelines.

## Materials and Methods

### Survey Areas

The survey was conducted in 17 prefectures in Japan (Aomori, Iwate, Akita, Miyagi, Yamagata, Fukushima, Ibaraki, Tochigi, Gunma, Chiba, Saitama, Tokyo, Kanagawa, Niigata, Yamanashi, Nagano, and Shizuoka prefectures) designated as target local governments in the “Concepts of Inspection Planning and the Establishment and Cancellation of Items and Areas to which Restriction on Distribution and/or Consumption of Foods concerned Applies” in March 24, 2017^10^^)^, March 23, 2018^11^^)^, and March 22, 2019^12^^)^. Based on the survey results of up to fiscal 2016^2^^,^^7^^,^^8^^)^, we focused on areas where the probability of r-Cs detection was considered to be high.

### Surveyed Foods

We surveyed all foods classified as general foods and originating from the survey areas, including fresh foods and processed foods. Based on the trends of r-Cs concentrations found in previous reports^2^^,^^7^^,^^8^^)^, we decided to focus on edible wild plants and wild mushroom as they’re difficult to control cultivation/feeding, and log- cultivated mushrooms. We also surveyed items that are controllable of cultivation/feeding to see if the inspection plans formulated by local governments were complied. Items of declining inspection frequency by fiscal yeasr, in comparison with fiscal 2016 (a year before revision of the guidelines) were chosen. The surveyed foods were purchased at retail stores and farmers’ markets of the surveyed areas, but the internet shopping sites were also used.

### Measurement of r-Cs Concentration

The assessment was done in accordance with the “Standard Operating Procedure for Sample Washing (Dirt Removal) for Tests of Radioactive Substances in Food” described in the Attachment to the “Testing Methods for Radioactive Substances in Food”^13^^)^. We washed fresh foods in water as necessary and used their edible parts as a sample for measurement. Samples of processed foods were prepared as in their original state for measurement. Each items was cut into sall pieces with a knife or similar instruments, then well-mixed, packed into a measurement container and used as a sample. Cutting boards, gloves, and other utensils were changed between samples to prevent contamination.

Samples were first screened by using a NaI(Tl) or CsI(Tl) scintillation spectrometer, except for samples of low packing density like dried products. The detailed screening process was described on r-Cs measureebt part of foods in the Attachment to “Concerning the Partial Revision of the Screening Method for Radioactive Cesium in Food Products”^14^^)^. The NaI(Tl) scintillation spectrometer (Aloka AccuFLEX γ7001) for screening process, a 20-ml vial was filled with sample material. The CsI(Tl) scintillation spectrometer (Techno-X FD-08Cs100) was designed to facilitate a 90-ml U8 container filled with sample material. Measurements was done for 60 min for both procedures. The range of energy measured was set at 540–830 keV for the NaI(Tl) scintillation spectrometer, 520–890 keV for the CsI(Tl) scintillation spectrometer for background counts. The instrumental conversion factors (Bq/cps) of the NaI(Tl) and CsI(Tl) scintillation spectrometers were determined at the beginning of each fiscal year by using a standard ^137^Cs solution. This performance confirmed that the measurement’s lower limit was 25 Bq/kg, and a screening level of 50 Bq/kg was achieved. For each set of measurements (around 20 samples), two blank samples and standard ^137^Cs solutions (25 Bq/kg and 50 Bq/kg) - or green tea leachate samples prepared to be 25 Bq/kg and 50 Bq/kg as previously determined with a high-purity germanium (HPGe) γ-spectrometer - were measured to assure no increase in the blank level, no change in the counts at the lower measurement limit/ screening level concentration, and no inconsistency in the energy scale.

Samples exceeding the measurement lower limit of 25 Bq/kg during the screening were subjected to confirmative inspection by using a HPGe γ-spectrometer (Canberra, GC4018 and GC4019). According to the method described in “The Series of Environmental Radioactivity Measuring Methods No. 7. Gamma-ray Spectrometry using Germanium Detector, September 2020 revision”^15^^)^, the weighted average activity concentration of ^134^Cs was measured. It’s gamma-rays emission of different decay energy were recorded at peaks of 475.4, 563.3, 569.3, 604.7, 795.8, 801.8, 1038.5, 1167.9, and 1365.1 keV. These values were usedf for calculated. This was added to the activity concentration of ^137^Cs measured at 661.6 keV to determine the r-Cs concentration in the sample. Measurement conditions were set where the sum of the respective detection limits for ^134^Cs and ^137^Cs would not exceed 20 Bq/kg, or the one-fifth of standard limit for general foods. Water was used as the base material for fresh foods. Ash was selected as the base material for dried foods. Effects of self-absorption were corrected on those values. Radioactive Cs concentrations were corrected for attenuation, with the date of sample purchase treated as the reference date. Confirmative inspection of dried products was conducted without screening because of the low packing density of dried products and the high measurement limit in the screening method. There are some general foods that are to be consumed afterbeing rehydrated. Their concentrations of r-Cs were converted to represent a soaked-state value by using the weight-change rate shown in the “Application of Testing Methods for Radioactive Substances in Food”^16^^)^. Conversion by using the weight-change rate was not performed for foods that are ingested without rehydration (e.g. dry powder-type items). Radioactive Cs concentrations were presented in two significant digits, according to the “Testing Methods for Radioactive Substances in Food” (Notice from the Director of the Food Safety Division, Pharmaceutical and Food Safety Bureau, Ministry of Health, Labour and Welfare, March 15, 2012)^13^^)^.

### Compilation of r-Cs Concentration Data

Foods subjected to inspection were classified into eight categories; edible wild plants, mushrooms, vegetables, beans and grains, fruits/nuts/seeds, aquatic products, meats including game meat/eggs/dairy products, and others (honey, seaweed, processed mulberry leaves, etc.). For each category, the concentration of r-Cs was calculated. Samples exceeding 25 Bq/kg of r-Cs concentration by confirmative inspection were categorized as “detected samples”. Those with more than 110 Bq/kg were categorized as “samples exceeding the standard limit”. The standard limit for r-Cs in general foods is 100 Bq/kg. The measurement results in this study are presented in two significant digits. Samples with an actual concentration of 105 Bq/kg or more in the confirmative inspection were described as “samples exceeding the standard limit”.

There were items in this study classified as “items for which cultivation/feeding control is difficult”. These samples were labeled as native, natural, wild, etc. For example, log-cultivated mushrooms were labeled as cultivated products and considered to required special concern upon the measurements because production material (a log) could be a possible source of radioactive materials. Edible wild plants and mushrooms were not cultivated products. The number of detected samples and the number of samples exceeding the standard limit were obtained, and labeled with their status of cultivation/feeding control.

## Results and Discussion

### Outline of r-Cs Survey Results

The total number of samples studied was 715 in fiscal 2017 (Table 1), 685 in fiscal 2018 (Table 2), and 683 in fiscal 2019 (Table 3). By category of surveyed items, mushrooms were most frequently surveyed items in all fiscal years. Folowing items were vegetables and edible wild plants in 2017, edible wild plants and vegetables in 2018 and 2019. Edible wild plants and mushrooms were considered to be food categories with high probability of exceeding the standard limit based on the results of previous surveys^2^^,^^7^^,^^8^^)^. Our study also focused on these items. The percentage of these food categories against the whole items surveyed was approximately 56% in fiscal 2017, 54% in fiscal 2018, and 53% in fiscal 2019. In contrast, the numbers of samples surveyed for aquatic products and others were only 3 items. This was because that the probability of exceeding the standard limit for these categories were low based on previous surveys^2^^,^^7^^,^^8^^)^. The proportion of each food category in the total samples inspected did not change substantially. However, in 2018 and 2019, the proportion of the vegetables category decreased slightly and the proportions of the fruits/nuts/seeds category and meat/eggs/dairy products category increased slightly compared to 2017. This is due to the fact that the items considered to be from natural origin, such as fruits/nuts/seeds and game meat were also actively surveyed, while the numbers of samples inspected by local governments were reduced in cultivation/feeding-controlable items such as fruits in comparison to fiscal 2016.

**Table 1.**
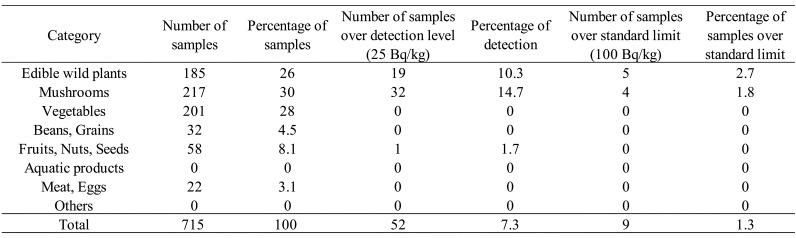
Results of the surveillance for radioactive cesium in fiscal year 2017

**Table 2.**
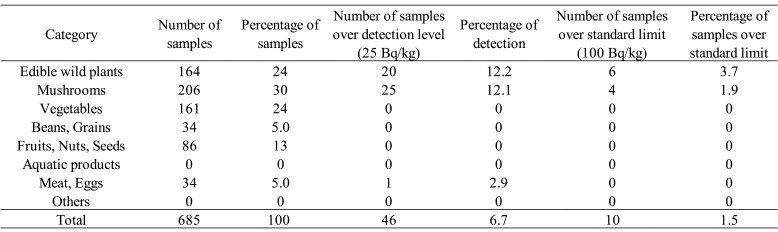
Results of the surveillance for radioactive cesium in fiscal year 2018

**Table 3.**
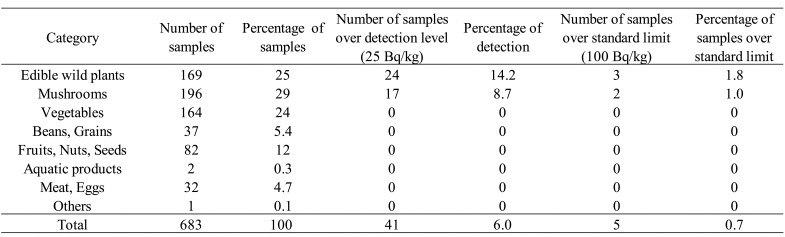
Results of the surveillance for radioactive cesium in fiscal year 2019

Detailed information on r-Cs detection in samples of “edible wild plant”, “mushroom”, “fruits, nuts and seeds”, and “meat, eggs, dairy products” in the confirmative inspection are shown in Tables 4, 5, 6, and 7, respectively. In the fiscal 2017 survey, r-Cs was detected in 52 of the 715 samples (7.3%). Nine samples (1.3%) exceeded the standard limit of 100 Bq/kg r-Cs in general foods (Table 1). Of the 9 samples exceeding 100 Bq/kg, 5 samples were edible wild plants (five koshiabura, Table 4) and 4 samples were mushrooms (3 log-cultivated shiitake and 1 koutake, Table 5). The r-Cs concentration detected ranged from 120 to 300 Bq/kg, with he highest concentration of 300 Bq/kg detected in koshiabura. Of 685 samples collected in fiscal 2018, 46 samples (6.7%) had detectable r-Cs, and 10 samples (1.5%) exceeded the standard limit (Table 2). Six samples from edible wild plants (4 koshiabura, 1 fatsia sprouts, and 1 bracken, Table 4) and 4 samples from mushrooms and their processed products (2 sakurashimeji, 1 shishitake, and 1 maitake powder, Table 5) exceeded the standard limit. The r-Cs concentrations ranged from 120 to 360 Bq/kg, with the highest concentration detected in bracken. In the fiscal 2019 survey, r-Cs was detected in 41 of 683 samples (6.0%), and 5 samples (0.7%) exceeded the standard limit (Table 3). Three samples from edible wild plants (3 koshiabura, Table 4) and 2 samples from mushrooms (1 koutake and 1 dried shiitake powder, Table 5) exceeded the standard limit. The r-Cs concentrations ranged from 110 to 260 Bq/kg. The highest concentration was detected in koshiabura.

**Table 4.**
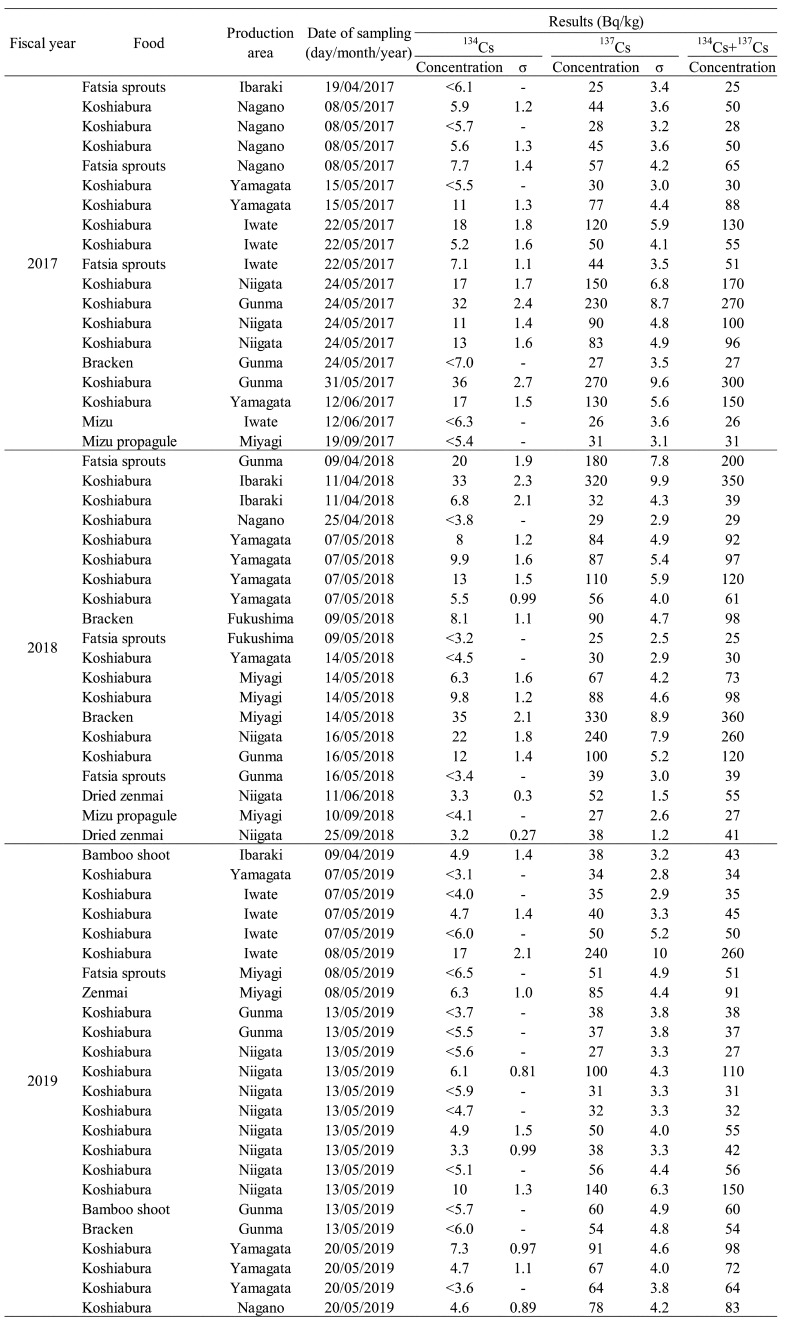
Results of the confirmative inspection of samples (edible wild plants) in which radioactive cesium was detected

**Table 5.**
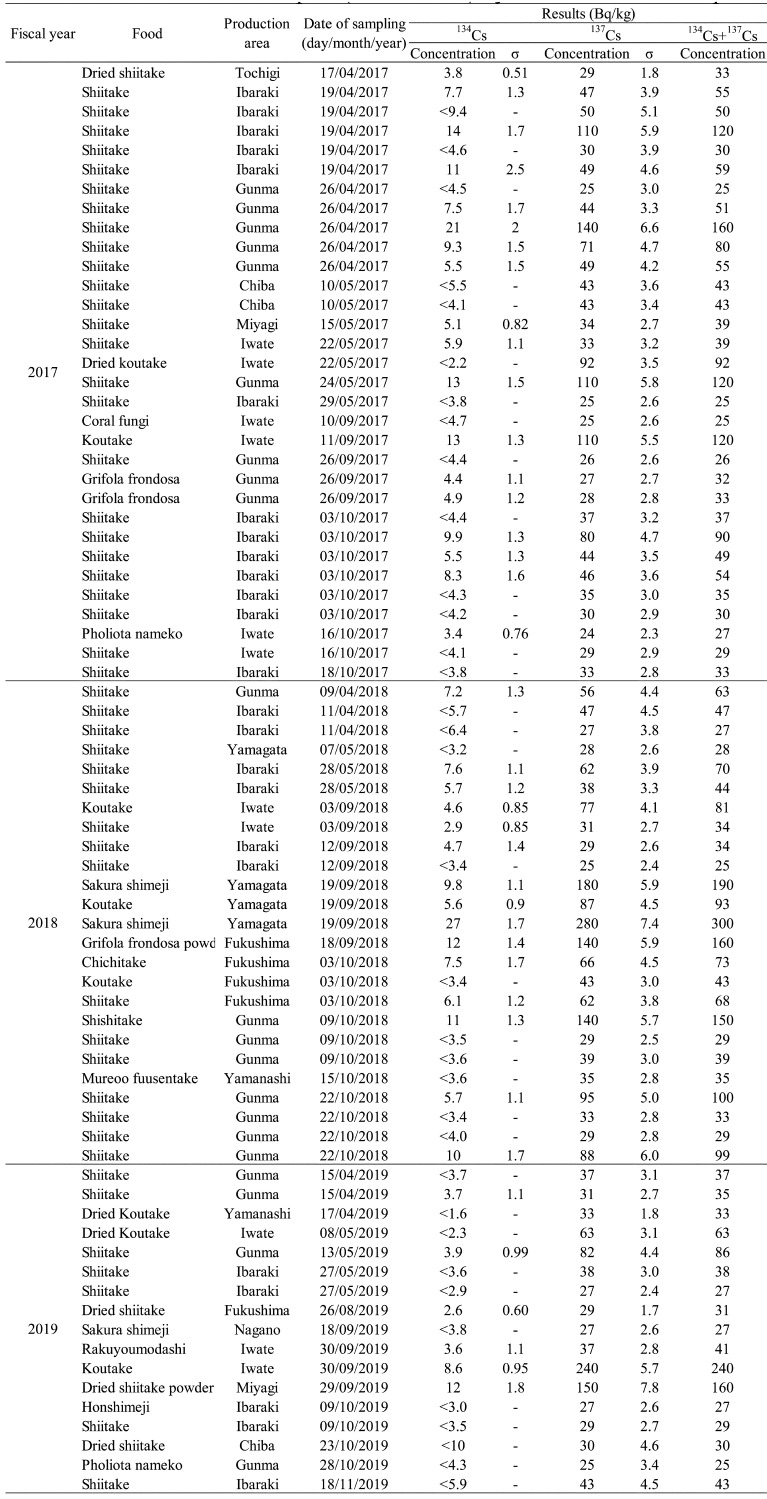
Results of the confirmative inspection of samples (mushrooms) in which radioactive cesium was detected

**Table 6.** Results of the confirmative inspection of samples (fruits, nuts and seeds, meat, eggs, dairy products) in which radioactive cesium was detected
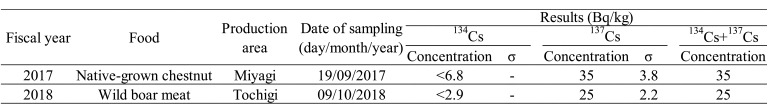

**Table 7.**
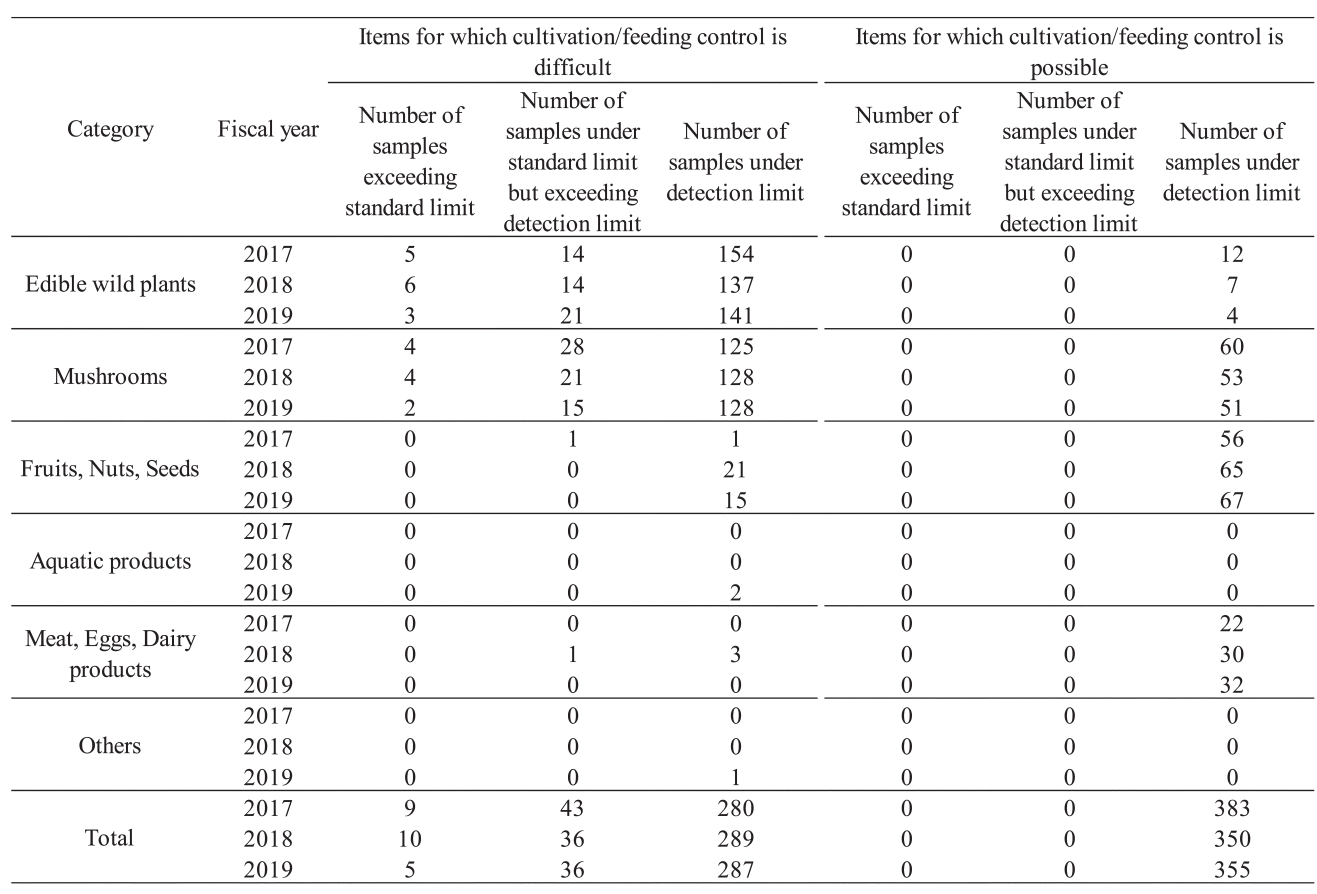
Results of surveillance of radioactive cesium by cultivation/feeding control status between fiscal 2017 and 2019

Although detection rates in this study were higher than rates previously reported in the fiscal 2012-2015 surveys (2.7-5.2%)^7^^,^^8^^)^, this does not necessarily indicate that radioactive contamination has spread, as this study focused on selected foods (mushrooms and edible wild plants) and areas considered to be with high probability of r-Cs detection. According to results of a nationwide inspection of radioactive materials in foods published on the MHLW website, the detection rate of r-Cs in all commercially-available foods was 0.54% in fiscal 2017^17^^)^, 0.49% in fiscal 2018^18^^)^, and 0.42% in fiscal 2019^19^^)^. These were lower than one-tenth the values observed in the present survey. The number of samples surveyed in this study was about 700, and only edible wild plants and mushrooms were found to be exceeding the standard limits. It should be noted, therefore, that the detection rate and the rate of exceeding the standard limits may have varied greatly depending on the availability of samples of edible wild plants and mushrooms.

### Comparison by Cultivation/Feeding Control Status

Table 7 summarizes results of the surveys for fiscal years on r-Cs concentrations in different food categories, devided by weather the items were in cultivation/feeding-controlable environment or less controllable environment. In each fiscal year, foods with cultivation/feeding controllable or difficult-to-control were examined in almost equal proportions. During the survey period, r-Cs was detected in edible wild plants (Table 4), mushrooms (Table 5), fruits/nuts/seeds (Table 6), and meat/eggs/dairy products (game meat) (Table 6), all of which were items for which cultivation/feeding control is difficult. They are so-called natural products, log-cultivated products, and game meat. It was reconfirmed that the concentration of r-Cs tended to be higher in the foods for which cultivation/feeding control is difficult, even when compared in the same food category. It should be noted, though, that the number of surveys on “fruits, nuts and seeds” and “meat, eggs, dairy products”, for which cultivation/feeding control is difficult, was small. In addition, this study focused on items such as koshiabura and koutake mushrooms, which were considered to have a particularly high probability of r-Cs detection among items from previous surveys. Their cultivation/feeding control is difficult. In fact, the detection rates of r-Cs for koshiabura were 62% in fiscal 2017, 50% in fiscal 2018, and 41% in fiscal 2019. The detection rates of r-Cs for koutake mushrooms were 100% throughout the study period. This explains the high detection rates and high rates of exceeding the standard limit found in the present study.

In contrast, r-Cs was detected in none of the total of 1,088 samples items, for which cultivation/feeding control is possible (Table 7). These results suggest that pre-shipment inspections by local governments were functioning effectively. We therefore consider that the reduction in number of items inspected by local governments for improving efficiency was appropriate, and that it was reasonable to reduce the extent of inspection of these items, for which cultivation/feeding control is plausible. This move is in accordance with the revised guidelines. Since efforts to reduce radioactive contamination at production sites are continuously implemented, the concentration of r-Cs in items, for which cultivation/feeding control is possible, is expected to remain low in the future.

### Edible Wild Plants

**Fig. 1.**
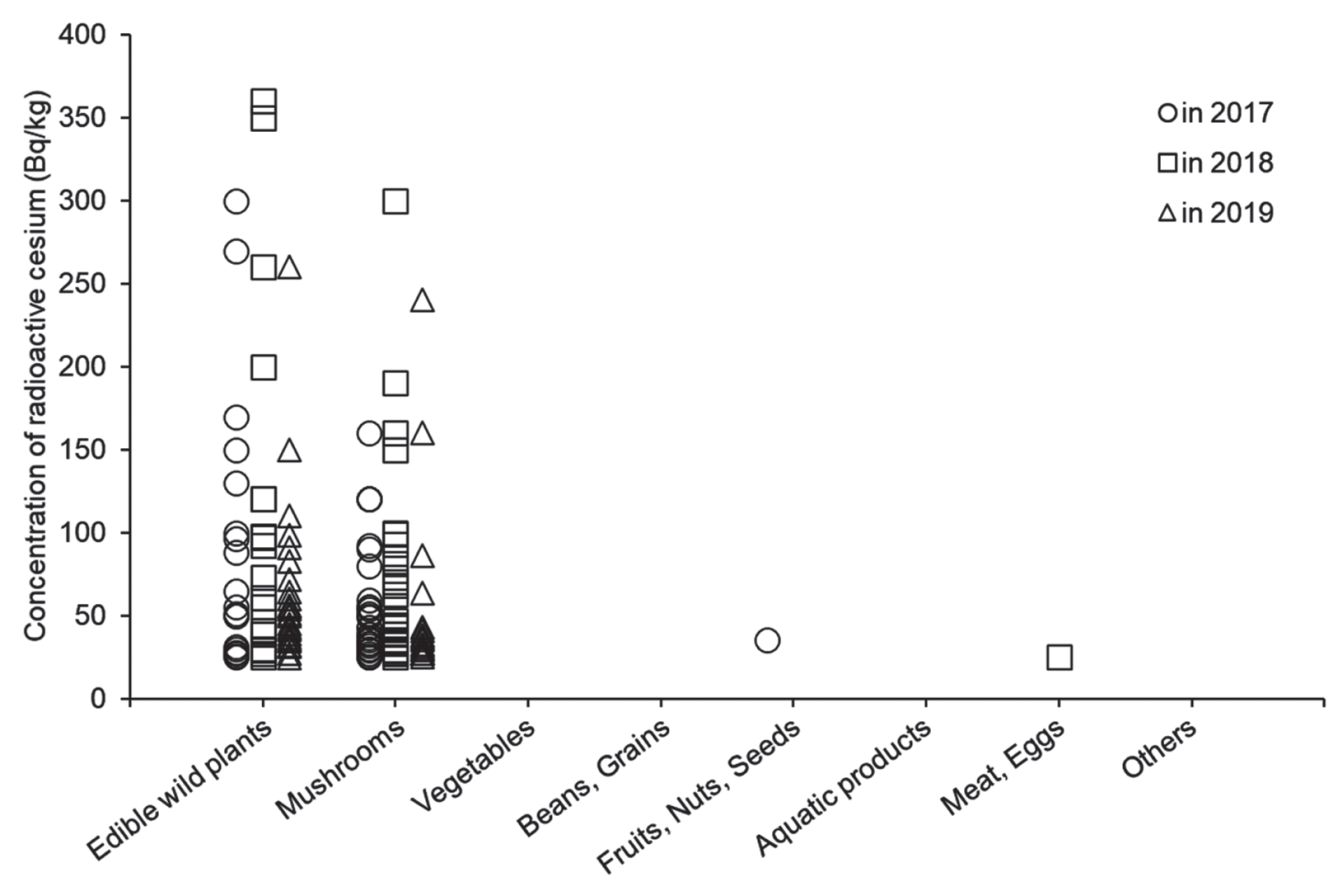
Distribution of radioactive cesium concentration in commercially-available foods surveyed in fiscal years 2017 (circle), 2018 (square), and 2019 (triangle).

For edible wild plants, r-Cs was detected in 19 of 185 samples (10.3%) in fiscal 2017, 20 of 164 samples (12.2%) in fiscal 2018, and 24 of 169 samples (14.2%) in fiscal 2019 (Tables 1-3). The types, production areas, date of purchase, and r-Cs concentrations of each edible wild plant sample with detectable r-Cs by final confirmative inspection are summarized in Table 4. The samples with detectable r-Cs were koshiabura, fatsia sprouts, zenmai, mizu propagule, bracken, bamboo shoot, and dried zenmai. These were presumed to be edible wild plants collected in the wild-environment. Throughout the study period, samples of koshiabura (12 samples), fatsia sprouts (1 sample), and bracken (1 sample) exceeded the standard limit, and the concentration of r-Cs ranged from 110 to 360 Bq/kg (Fig. 1). Radioactive Cs was detected in 44 out of 91 samples of koshiabura. Among them, 12 samples exceeded the standard limit. It has been reported that koshiabura has a characteristic of absorbing Cs easily^20^^,^^21^^)^, thus, it was not surprising that relatively high concentrations of r-Cs were found in koshiabura at the present survey. On the other hand, 23 samples of edible wild plants confirmed to be cultivated products were surveyed, but r-Cs was not detected in any of them (Table 7).

### Mushrooms

Mashroom samples with detectable r-Cs accounted for 32 out of 217 samples (14.7%) in fiscal 2017, 25 of 206 samples (12.1%) in fiscal 2018, and 17 of 196 samples (8.7%) in fiscal 2019 (Tables 1-3). The types, production areas, date of purchase, and r-Cs concentrations of each mushroom sample with detectable r-Cs by the confirmative inspection are summarized in Table 5. Throughout the study period, 10 samples exceeded the standard limit. Mashroom r-Cs concentrations ablve the standard limit ranged from 120 to 300 Bq/kg (Fig. 1). Some of the mushroom samples that exceeded the standard limit were dried powder of log-cultivated mushrooms. The measurement results of dry powder samples were not subjected to weight-conversion for wet state simulation, because such powder is ingested without rehydration in some occations. In addition, concentrations in dried products may become higher than their oginal materials because of the condensing-nature of drying process, thus making it more likely to exceed the standard limit. When interpreting such processed foods’ results, it is important to pay attention to the control of raw materials’ r-Cs concentrations. The mushrooms with detectable r-Cs were natural mushrooms, therefore, it was presumed that the material were collection in the wild-habitat environment. Log-cultivated mushrooms were also prone to influence of radioactive materials, when firmed in large scale production. It is considered that the r-Cs concentrations in these mushrooms were still in high concentration range compared to other foods. In the mean time, r-Cs was non-detectable in 164 samples of fungal bed-cultivated mushrooms, which were considered to be easy to control the cultivation/feeding condition. The type of mushroom with the largest sample size was shiitake (including dried products), accounting for 65% of all mushrooms. Radioactive Cs was detected in 52 out of 404 shiitake samples, and 4 samples exceeded the standard limit. All the shiitake samples with detectable r-Cs concentrations were those grown on logs. Based on the above results, we consider that the surveys focused on natural mushrooms and log-cultivated mushrooms are effective strategies to improve the efficiency of radioactive Cs inspections, as these items are grown in condition where cultivation/feeding control is difficult.

### Fruits, Nuts, and Seeds

The sample size of fruits, nuts, and seeds in the surveys were 58 samples for fiscal 2017, 86 samples for fiscal 2018, and 82 samples for fiscal 2019 (Tables 1-3). In the 2017 survey, 35 Bq/kg of r-Cs was detected by the confirmative inspection in one chestnut sample that was likely to be collected in the wild (Table 6). No sample had detectable r-Cs in fiscal 2018 and 2019 surveys. In past, r-Cs concentration in chestnuts sometimes exceeded 25 Bq/kg, but had not exceeded the standard limit^7^^,^^8^^)^. Although measures to reduce the amount of r-Cs have been taken at production sites of cultivated chestnut trees and fruit trees by high-pressure washing and pruning, it is difficult to take such measures for native chestnut and fruit trees in the wild. In the future, it will be necessary to carefully monitor natural, wild and native fruits, nuts, and seeds, for which cultivation/feeding control is difficult.

### Meat and Eggs

Meat and eggs sample sizes in the surveys were 22 samples for fiscal 2017, 34 samples forfiscal 2018, and 32 samples for fiscal 2019. Although r-Cs was detected by the confirmative inspection in 1 wild boar meat sample in the 2018 survey (Table 6), no sample has exceeded the standard limit throughout the survey period. It is difficult to control wild birds and other animals living in wild habitat including mountains and fields. This pragmatic aspect might present coherency with the results of r-Cs concentrations exceeding the standard limit in these types of samples in past surveys. The current proportion of commercial game meat in the market is small, but we need to take into account that the government is now promoting the use of game meat (gibier)^22^^)^. These game meat products that are circulating in supply chain is expected to increase. It is preferable that the game meat that exceed the r-Cs standard limit to be excluded from the commercial distribution, thus, it is important for local governments to take measures such as all-product inspection in these animal and bird products before shipment^23^^,^^24^^,^^25^^,^^26^^)^. At the same time, the surveys examined samples of animals and animal-derived products that are considered to be easy to control their original animals’ cultivation/feeding condition. Included in this study are 84 samples period from beef, pork, chicken, horsemeat, eggs, among others in this survey. Results showed that no r-Cs was detected. This suggests that r-Cs concentrations in meat and eggs, when thery are from controlled feeding environment, continued to be low, and this is most likely to do with the appropriate management.

### Vegetables, Beans, and Cereals

During the study period, a total of 629 samples of vegetables, beans, and cereals were examined. No sample was with detectable r-Cs concentrations. All of these food categories consist of items where cultivation/feeding control is possible, therefore, we consider that r-Cs concentration was appropriately controlled. Probability of these food categories to have detectable r-Cs in the future appears to be low. Reduction of inspection frequency in these food categories is appropriate to optimal balance of detection with a certain measurement resources .

### Aquatic Products

This study included only few surveys on aquatic products. This is because the past results showed the probability of detecting r-Cs in commercial circulated aquatic products to be not high. Two samples of processed freshwater products were surveyed in fiscal 2019, and no r-Cs was detected. Natural aquatic products fall into the category of items, for which cultivation/feeding control is difficult. However, according to the results of nationwide radioactive material inspections of foods that were published on the MHLW website, a total of 4,683 samples of commercial aquatic products (excluding seaweed) were inspected from fiscal 2017 to 2019, and the number of samples with r-Cs detection was only 2 samples in 2017 and 1 sample in 2018, with none of these samples exceeding the standard limit^17^^,^^18^^,^^19^^)^. Since the amount of commercial natural freshwater products is small, there is some difficulty in obtaining samples for survey. Nevertheless, according to results of inspections of non-commercial products including pre-shipment inspections, more than 95% of the aquatic products with r-Cs detection were freshwater fish and shellfish^17^^,^^18^^,^^19^^)^. It is therefore necessary to pay attention to the r-Cs concentration in freshwater products from natural habitat.

In this study, we purchased commercially-available foods, focusing on production areas and items with high probability of r-Cs detection. The surveyed r-Cs concentrations were from 715, 685, and 683 samples in fiscal years 2017, 2018, and 2019, respectively. The proportion of samples that exceeded the standard limit for r-Cs in general foods (100 Bq/kg) was 1.3% in fiscal 2017, 1.5% in fiscal 2018, and 0.7% in fiscal 2019. All the samples with r-Cs detection (25 Bq/kg or more), as well as the samples that exceeded the standard limit, were foods of difficult-to-control cultivation/feeding origin. Although this study focused on foods with high probability of r-Cs detection, the rate of samples exceeding the standard limit remained sufficiently low, suggesting that the pre-shipment inspection systems of local governments were functioning properly. Even though we investigated the concentration of r-Cs in foods for which the extent of inspection was reduced due to the revised guidelines, no r-Cs was detected in any of those samples. We therefore suggest that the items were appropriately selected when reducing the test sample selection. On the other hand, r-Cs concentrations in edible wild plants, natural mushrooms, and log-cultivated mushrooms remained high. Therefore, we suggest that it is necessary to continue intensive inspections and surveys for these and other items for which cultivation/feeding control is difficult.
